# Investigating the Role of the Sinus Microbiome and Cytokine Profile in the SNOT-22 Response After Functional Endoscopic Sinus Surgery in Chronic Rhinosinusitis Patients

**DOI:** 10.3390/jcm14134446

**Published:** 2025-06-23

**Authors:** David Hoying, Naseer Sangwan, Mohamad R. Chaaban

**Affiliations:** 1 School of Medicine, Case Western Reserve University, Cleveland, OH 44106, USA; hoyingdr@gmail.com; 2 Head and Neck Institute, Cleveland Clinic, Cleveland, OH 44195, USA; sangwan@ccf.org; 3 Department of Inflammation and Immunity, Lerner Research Institute, Cleveland Clinic, Cleveland, OH 44195, USA

**Keywords:** SNOT-22, chronic rhinosinusitis, FESS, bacteriology

## Abstract

**Background**: Functional endoscopic sinus surgery (FESS) is the treatment of choice for medically refractory CRS. However, the success rate of FESS is dependent on both baseline medical and demographic characteristics. Consequently, we performed an analysis of systemic/nasal cytokines and the sinus microbiome to assess their impact on the SNOT-22 response after functional endoscopic sinus surgery (FESS). **Methods**: A prospective observational study was performed on 44 patients with chronic rhinosinusitis undergoing FESS between December 2021 and September 2022. Diseased sinus tissue from 25 patients was subjected to whole-exome sequencing (WES) for taxonomical profiling of the sinus bacterial composition. Additional data collection included demographics, comorbidities, baseline sinonasal outcome test scores, post-operative sinonasal outcome test scores (at 3–4 months), and nasal/systemic cytokines. **Results**: Our analysis demonstrated that CRSwNP patients in the surgical responder cohort had statistically significantly higher median [P25, P75] levels of intra-nasal IL-5, indicating type 2 sinonasal disease (63 pg/μL [28, 118] versus 17 pg/μL [16.6, 18], *p* = 0.04). At the genus level, the relative abundance of *Staphylococcus* was significantly higher in the surgical non-responder cohort compared to the responder group. An ROC curve was highly accurate at distinguishing responders versus non-responders to FESS based on a microbiota-based random forest model (AUC = 0.92). **Conclusions**: Intra-nasal IL-5 levels and the bacterial composition of the sinus microbiome may be important predictors of symptomatic response after sinus surgery.

## 1. Introduction

Functional endoscopic sinus surgery (FESS) is the primary treatment choice for medically refractory chronic rhinosinusitis (CRS), a prevalent inflammatory disorder affecting the paranasal sinuses and nasal passages. Despite its established efficacy, the clinical outcomes of FESS can exhibit heterogeneity, with a success rate of 80% to 98% [1,2,3,4]. Several clinical and demographic factors have been investigated as potential predictors of quality-of-life improvement after FESS, including asthma, aspirin intolerance, prior sinus surgery, prior polypectomy, allergies, smoking, age, and gender [4,5,6,7,8,9,10]. The cytokine profile and sinus microbiome are two potential factors that may also contribute to the FESS response rate, requiring further investigation.

The inflammatory profile of CRS is predominantly categorized as non-type 2 (Th2) inflammation and Th2 inflammation [11]. The impact of the patient’s nasal inflammatory profile on surgical failure has been controversial. Some studies have reported that Th1 and Th3 inflammation are associated with worse surgical outcomes [12,13]. Alternatively, several studies have also shown that an eosinophilic-predominant response, Th2 inflammation, and eosinophilia are correlated with poor surgical response, defined predominantly by recurrence [14,15,16,17]. As a result of these conflicting results, and with a focus on recurrence patterns, further research is needed to better define how the inflammatory profile of CRS impacts the quality-of-life score response after sinus surgery.

There is limited research available on how the sinus microbiome affects response to treatment in CRS. It has been shown that increases in *Acinetobacter* spp. post-operatively are associated with improved response after sinus surgery due to their anti-inflammatory effects [18]. Consequently, this raises important questions about how baseline bacterial composition may be predictive of symptomatic improvement after FESS.

The objective of this study was to assess the impact of the baseline relative abundance of intra-sinonasal bacteria and the cytokine profile on symptomatic response to treatment in patients with CRS after functional endoscopic sinus surgery.

## 2. Methods and Materials

The study was conducted in accordance with the Declaration of Helsinki and approved by the Institutional Review Board of the Cleveland Clinic (protocol #21-883, 22 October 2021). In this prospective cohort study, we included patients with chronic rhinosinusitis (CRS) undergoing functional endoscopic sinus surgery (FESS) between December 2021 and September 2022. Any patient undergoing FESS with at least bilateral total ethmoid disease on a sinus CT scan was included in this study. Patients were excluded if they presented one of the following characteristics: unilateral CRS disease, receipt of immunotherapy or biologics within 6 months of enrollment, receipt of oral steroids within the 4 weeks preceding enrollment, or a diagnosis of cystic fibrosis, sinus tumors, immunodeficiency, or ciliary dyskinesia. We collected data on the following clinical and demographic characteristics: body mass index (BMI), comorbidities, baseline clinical severity evaluated using the sinonasal outcome test (SNOT-22) score, baseline measurements of local and systemic alarmins/T-helper cytokines, and post-operative SNOT-22 scores at the end of the 3–4-month follow-up period.

### 2.1. CRS Clinical Severity

The clinical severity of CRS was determined utilizing the validated questionnaire SNOT-22 [19]. CRS disease burden has been determined to be best approximated by patient-reported symptomology, which makes SNOT-22 a valuable tool for assessing quality of life [20]. The questionnaire consists of 22 questions about relevant clinical symptoms, each graded on a scale of 0 to 5, with 5 indicating the most severe symptoms and 0 representing no reported issue with the specific symptom.

### 2.2. Nasal and Systemic Cytokine Analysis

We collected baseline nasal and systemic cytokine levels in many patients undergoing FESS. The systemic cytokine analysis was performed on blood obtained during the surgical procedure. Collection of samples for nasal cytokine analysis was performed utilizing a previously published protocol [21]. In this approach, a polyvinyl alcohol (PVA) sponge was placed in the middle meatus for 10 min. The sponge was then removed from the middle meatus and centrifuged. The collected supernatant following centrifugation was then stored at 80 °C for subsequent cytokine analysis. Cytokine analysis was performed utilizing the Human Cytokine/Chemokine/Growth Factor 71-Plex Clinical (RUO) Assay (HD71-CLIN) from Eve Technologies (Calgary, AB, Canada).

In the cytokine analysis, the responder cohort was defined as patients with at least a 50% reduction in baseline SNOT-22 score up to 4 months after FESS. Patients who did not meet this cut-off were classified as non-responders.

### 2.3. Sinus Microbiome Analysis

DNA was extracted from sinus tissue samples using a DNeasy PowerLyzer PowerSoil kit (Qiagen, PL Venlo, The Netherlands) following the manufacturer’s guidelines. Metagenomic DNA samples were quantified by Quant-iT PicoGreen dsDNA Assay (Life Technologies, Carlsbad, CA, USA) and standardized to a concentration of 50 pg/μL. Illumina sequencing libraries were then generated from 100–250 pg of normalized DNA utilizing a Nextera XT DNA Library Preparation kit (Illumina, San Diego, CA, USA), adhering to the manufacturer’s recommended protocol, with scaled volumes as appropriate. Before sequencing, libraries were pooled by collecting equal volumes (200 nL) of each library from batches of 96 samples. To assess the insert sizes and concentrations of these pooled libraries, an Agilent Bioanalyzer DNA 1000 kit (Agilent Technologies, San Diego, CA, USA) was employed. Subsequent sequencing was carried out on a NovaSeq 6000 platform using the SP1 kit option. Following sequencing, rigorous post-sequencing quality control procedures, including read trimming and filtering, were performed using the Nesoni software tool (v0.134) (https://github.com/Victorian-Bioinformatics-Consortium/nesoni, accessed on 20 July 2024). High-quality reads were aligned against the human genome (Gencode version GRCh38) using STAR aligner (v2.7.11b). Reads aligned to the human genome were excluded from subsequent analyses. For taxonomical analysis, non-human reads underwent processing utilizing MetaPhlAn3, a tool known for its proficiency in profiling microbial communities through metagenomic shotgun sequencing data. To elucidate functional attributes, the HUMAN3 resource was harnessed to annotate and interpret the potential functional roles associated with non-human reads [22].

For receiver operating characteristic (ROC) curve analysis, feature selection was performed using the Boruta algorithm with maxRuns set to 300 and a *p*-value threshold of 0.05. The dataset was then randomly split into equal training and test sets. A random forest model was constructed using the randomForest package in R (version 4.7-1.2), with 1000 trees (ntree) and a tune length of 25 for optimizing the mtry parameter. The model used default settings for node size (1 for classification or 5 for regression) and maximum number of terminal nodes (unlimited). K-fold cross-validation (k = 10) was employed during training to ensure robust performance estimation. The model’s performance was evaluated on the test set using appropriate metrics (e.g., accuracy, precision, recall, and F1-score for classification; MSE and R-squared for regression). Variable importance was assessed using the mean decrease in accuracy or Gini impurity.

For the sinus microbiome analysis, a minimum clinically important difference (MCID) for total SNOT-22 of 8.9 or greater was used to stratify cohorts into responder and non-responder cohorts up to 4 months after FESS [23].

### 2.4. Statistical Analysis

Differences between categorical variables in the non-responder and responder cohorts were assessed by Pearson’s chi-square or Fisher’s exact test, depending on the sample size. Continuous variables, including cytokine levels, were evaluated for differences between groups by the Wilcoxon Rank Sum test. Cytokine levels are reported as median [25th percentile, 75th percentile]. The receiver operating characteristic (ROC) curve distinguished responders versus non-responders to FESS based on a microbiota-based random forest model. All statistical analyses were conducted using SAS version 9.4 (The SAS Institute, Cary, NC, USA), and a *p*-value less than 0.05 was considered statistically significant. Due to the small sample size within the subgroups of the cytokine analysis, particularly the CRSwNP cohort, we performed a sample size calculation to estimate the number of patients required to detect a clinically meaningful difference in IL-5 between responders and non-responders. Based on observed variability (estimated pooled SD = 12.07 pg/μL) and a target difference of 5 pg/μL, we estimate that approximately 186 patients (93 per group) would be needed to achieve 80% power with a two-sided alpha of 0.05. We opted to calculate the required sample size rather than post hoc power, as the latter is known to offer limited interpretive value once study results are available. Additionally, given the small sample size and the exploratory nature of this study, we conducted cytokine comparisons using a complete case approach. We did not perform imputation, as this would have required strong assumptions about the missing data mechanism and could have introduced additional bias.

## 3. Results

### 3.1. Cytokine Analysis

There were a total of 44 patients with follow-up SNOT-22 scores after functional endoscopic sinus surgery (FESS). Of the 44 patients with CRS, 23 were categorized as non-responders (52.3%) and 21 as responders (47.7%). Table 1 displays the demographics, comorbidities, and baseline systemic/nasal cytokine profile of patients with CRS who underwent FESS. There was no statistical difference in age, BMI, race, gender, smoking status, asthma diagnosis, allergic rhinitis diagnosis, aspirin-exacerbated respiratory disease (AERD) diagnosis, or nasal/systemic cytokine profile between the responder and non-responder cohorts when including both clinical phenotypes of CRS. Patients with CRSwNP (n = 16, 62%) were more likely to respond to FESS, defined as a 50% reduction in post-operative SNOT-22 score compared to baseline SNOT-22 score, compared to patients with CRSsNP (n = 5, 28%) (*p* = 0.03).

We then stratified the CRS cohort into the predominant clinical phenotypes of CRSwNP and CRSsNP. Of the 26 patients with CRSwNP, 10 were classified as non-responders (38%) and 16 as responders (62%). There was no statistical difference in demographics, comorbidities, and systemic cytokines between responders and non-responders in the CRSwNP cohort. CRSwNP patients that responded to FESS were determined to have a statistically significantly higher median level of nasal IL-5 compared to non-responders (63 pg/μL [28, 118] versus 17 pg/μL [16.6, 18], *p* = 0.04). Table 2 summarizes the baseline characteristics and cytokine comparisons within the CRSwNP subgroup. No other nasal cytokines differed significantly. In contrast, the CRSsNP subgroup (n = 18) showed no statistically significant differences between responders and non-responders in demographics, comorbidities, or nasal/systemic cytokine levels. These findings are detailed in Table 3. Supplementary Tables S1–S3 report the number of missing patients within each subgroup for the cytokine analysis, due to cytokine data being unavailable for the entire cohort.

#### Sinus Microbiome Analysis

**Figure 1:**
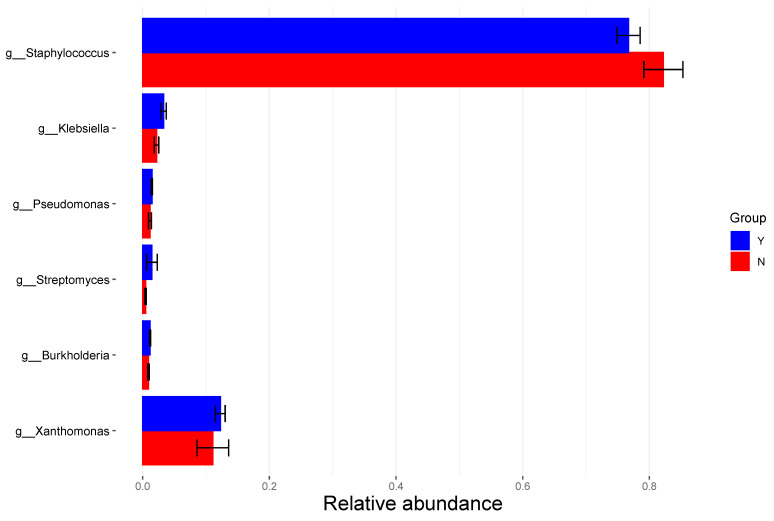
Relative abundance at the genus level of microbial species in the sinus microbiome, stratified by response to treatment. Key: Y: responder, N: non-responder.

**Figure 2:**
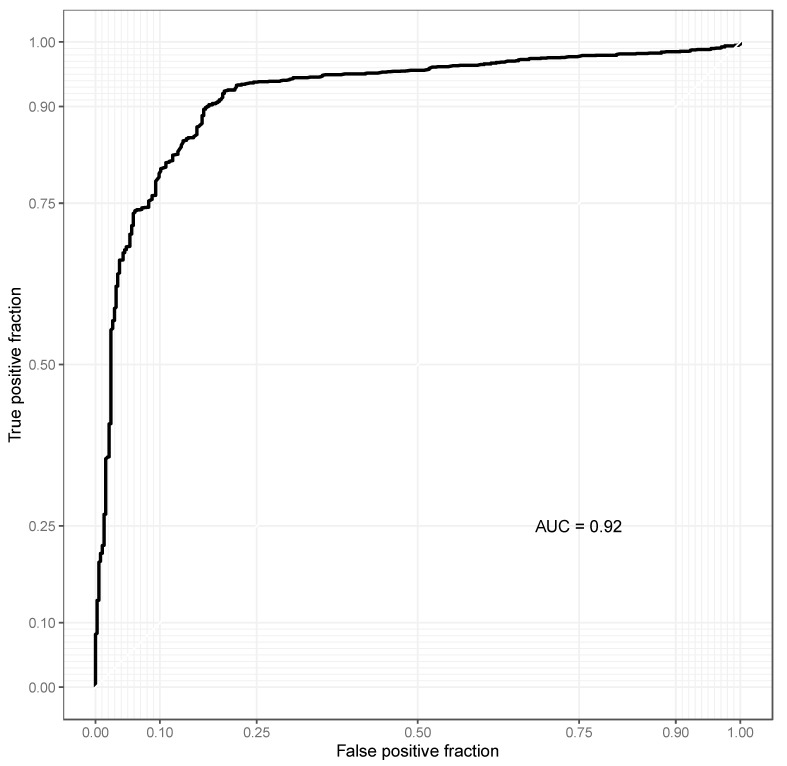
Receiver operating characteristic (ROC) curve distinguishing responders versus non-responders to FESS based on a microbiota-based random forest model.

Among the 44 patients with follow-up SNOT-22 scores, the fresh frozen sinus mucosa removed during FESS of 29 patients underwent taxonomic profiling of the bacterial composition. In the cohort of 29 CRS patients, there were 5 patients with sub-optimal clinical response (2 CRSsNP; 3 CRSwNP) and 24 patients with a successful post-operative response (5 CRSsNP; 19 CRSwNP). At the genus level, the relative abundance of *Staphylococcus* was highest in both the optimal and sub-optimal responsive cohorts, followed by *Xanthomonas* and *Klebsiella* (Figure 1). However, non-responders showed greater enrichment of Staphylococcus, while responders had increased abundance of Klebsiella and Streptomyces (Figure 2). The bacterial composition of the sinus microbiome also comprised *Pseudomonas*, *Streptomyces*, and *Burkholderia*. A notable difference observed between the responder and non-responder cohorts was the increased enrichment of *Staphylococcus* in the non-responder cohort. Patients with an adequate response to FESS appeared to instead have increased enrichment of *Klebsiella* and *Streptomyces* compared to patients without a sufficient response. An ROC curve (Figure 2) was highly accurate at distinguishing responders versus non-responders to FESS based on a microbiota-based random forest model (AUC = 0.92).

## 4. Discussion

The aim of this study was to assess the association of the systemic/nasal cytokine profile and the sinus microbiome with FESS symptomatic treatment response. Our analysis is one of the first in the literature to assess the association of the baseline sinus microbiome with FESS treatment response in CRS patients. We identified that systemic and nasal cytokine levels did not differ significantly when stratifying patients by a 50% reduction in baseline SNOT-22 score after sinus surgery. However, patients in the CRSwNP cohort that were classified as responders had statistically significantly higher levels of intra-nasal IL-5 compared to non-responders. When considering clinical factors associated with FESS symptomatic response, patients with CRSwNP were more likely to demonstrate symptomatic improvement post-operatively compared to patients with CRSsNP. Our evaluation of the sinus microbiome revealed that the largest difference between responders’ and non-responders’ microbial composition was increased enrichment of *Staphylococcus* in the non-responder cohort. An ROC curve was employed to assess the accuracy of distinguishing responders versus non-responders to FESS based on a microbiota-based random forest model, which was demonstrated to be a highly accurate prediction model.

The only clinical characteristic observed to be associated with symptomatic surgical response was the presence of nasal polyps. In this study, CRS patients with nasal polyps had significantly higher rates of adequate reduction in the total SNOT-22 score. Our findings are further supported by a prospective observation study that reported that symptomatic/quality-of-life improvement was significantly greater in CRSwNP patients compared to CRSsNP patients for general health, vitality, social function, nasal obstruction, and altered sense of smell [24].

Overall, the symptomatic improvement measured by the total SNOT-22 score reduction after FESS was relatively independent of systemic and nasal cytokine levels. When stratifying by CRS with nasal polyps, elevated levels of nasal IL-5 were associated with increased rates of response. A study of the baseline mucus cytokines of 147 patients undergoing FESS supports our findings by reporting that IL-5 was an independent predictor of post-operative total SNOT-22 and domain score improvement [25]. IL-5 has a critical role in the pathophysiology of CRSwNP due to its ability to promote dedifferentiation, migration, activation, and survival of eosinophils [26]. Previous research has indicated that eosinophils are involved in the pathophysiology of nasal polyp recurrence and failure of FESS [15,27]. Chowdhury et al. speculated that baseline levels of intra-nasal IL-5 may be correlated with the severity of polyp disease, requiring sinus surgery for symptomatic relief [25]. This aligns with our findings that patients with elevated baseline intra-nasal IL-5 had greater symptomatic improvement.

The microbial composition of the sinus microbiome has become an increasingly studied area to define its role in the development and severity of CRS. The bacterial profile of the healthy sinus is commonly enriched with *Staphylococcus*, *Corynebacterium*, *Peptoniphilus*, and *Propionibacterium* [28,29,30,31,32]. Alteration of the healthy sinus microbiome can allow opportunistic pathogens to proliferate, contributing to disease development [32]. Previous research has shown that there is significant inter-individual variation in the microbial composition of the sinus microbiome between CRS patients and healthy controls [33]. Our analysis showed that the non-responder cohort had increased enrichment of *Staphylococcus* at the genus level compared to the responder cohort, which could indicate that the sinonasal bacterial profile is associated with treatment response after FESS. Similar to our findings, a retrospective study of 24 medically refractory patients undergoing FESS identified that *S. aureus* biofilms were associated with severe and surgically resistant disease [34]. Although not statistically significant, a study by Ramakrishnan et al. demonstrated that *Staphylococcus aureus* was 3-fold higher in patients with a poorer surgical response, which further supports the findings of this present study [32]. We also identified that the relative abundance of *Klebsiella* was decreased in the non-responder cohort compared to the responder group. There appears to be limited data available describing the presence of *Klebsiella* species in the sinus microbiome, which may be an area for future research.

The most frequently reported bacteria identified within the sinus microbiome of patients with CRS are *Staphylococcus*, *Propionibacterium*, and *Corynebacterium* at the genus level; however, these bacteria are also highly prevalent in the healthy sinus microbiome [35]. Previous studies have been unable to consistently identify a single microbe associated with CRS. Increased relative abundance of *C. tuberculostearicum* and *C. accolens* has been reported in CRS patients by two distinct studies [36,37]. Alternatively, many species have been reported to be found at lower relative abundance across several studies, including *Prevotella* spp., *Lactobacillus* spp., *Peptoniphilus* spp., *Propionibacterium acnes, Acinetobacter johnsonii,* and *Corynebacterium confusum* [18,37,38,39]. Our analysis of the bacterial profile of patients with CRS did not demonstrate a significant abundance of species within these genera. While the bacterial composition of the sinus microbiome in CRS patients has been well-researched, there appears to be limited data available on how the baseline sinus microbiome may impact treatment response. However, a study by Cleland et al. identified that the relative abundance of *Acinetobacter* spp. was increased significantly after sinus surgery compared to control subjects in patients who responded well to FESS [18]. An additional study by Ramakrishnan et al. identified a baseline sinus microbiome prior to FESS, with an increased abundance of Actinobacteria associated with optimal surgical response [31]. Our study did not assess the microbiome post-operatively, which prevents us from demonstrating if our responder cohort experienced similar microbial changes. Although our sample size is small, our ROC curve shows a highly accurate model for predicting sinus surgery symptomatic response based on a microbiota-based random forest model. This demonstrates the importance of further research in this area to better understand how the baseline microbial composition may affect symptomatic response after FESS.

There are several limitations to this analysis. Our sample sizes are small, limiting generalization of this data beyond our cohort. Our study’s scope was restricted to CRS patients who sought treatment at our Cleveland Clinic rhinology clinic. This could introduce bias in participant selection and potentially limit the ability to apply our findings to all CRS patients nationwide in the U.S. Our collection of systemic and local sinonasal cytokines was limited to a single time point, preventing us from assessing their changes over time or post-sinus surgery. Furthermore, we were unable to establish correlations with post-operative sinonasal-specific quality of life scores using tools like SNOT-22. Additionally, data was not available for each patient within our cytokine analysis, further reducing the sample size. This may have limited the power to identify statistically significant differences between the responder and non-responder cohorts. We were also unable to complete the microbiome analysis for all 44 patients included in this study due to limitations in sample collection. This resulted in a small cohort for our non-responder group within the microbiome analysis. Furthermore, the limitation of using PCR (rather than swabs) resulted in a lack of knowledge regarding whether a given species was alive at the time of the study.

## 5. Conclusions

Our analysis demonstrates that increased levels of intra-nasal IL-5 are associated with better symptomatic improvement after sinus surgery. Additionally, we identified that decreased relative abundance of *Staphylococcus* spp. may be associated with improved symptomatic relief after functional endoscopic sinus surgery. Future research is needed to better understand the interplay between the microbial composition and the intra-nasal cytokine profile, as well as further investigate how the sinus microbiome may be used to predict FESS response in otolaryngology clinical practice.

## Figures and Tables

**Table 1:** Differences in predictors between responder versus non-responder patients with chronic rhinosinusitis.

Factor	Total (N = 44)	Non-Responder (N = 23)	Responder (N = 21)	*p*-Value
Age (years)	54.0 [37.5, 66.5]	60.0 [37.0, 67.0]	53.0 [42.0, 63.0]	0.44 ^b^
BMI (kg/m^2^)	29.0 [26.2, 36.1]	28.3 [26.5, 37.3]	29.0 [24.8, 35.1]	0.75 ^b^
Race *				0.99 ^d^
Non-White	5 (11.9)	3 (13.6)	2 (10.0)	
White	37 (88.1)	19 (86.4)	18 (90.0)	
Gender				0.97 ^c^
Male	25 (56.8)	13 (56.5)	12 (57.1)	
Female	19 (43.2)	10 (43.5)	9 (42.9)	
CRS Phenotype				***0.027 ^c^***
Without Polyps	18 (40.9)	13 (56.5)	5 (23.8)	
With Polyps	26 (59.1)	10 (43.5)	16 (76.2)	
Type of Surgery				0.86 ^c^
Primary	33 (75.0)	17 (73.9)	16 (76.2)	
Revision	11 (25.0)	6 (26.1)	5 (23.8)	
Smoking Status				0.69 ^c^
Never	28 (63.6)	14 (60.9)	14 (66.7)	
Current or Former	16 (36.4)	9 (39.1)	7 (33.3)	
Asthma Present				0.098 ^c^
No	16 (36.4)	11 (47.8)	5 (23.8)	
Yes	28 (63.6)	12 (52.2)	16 (76.2)	
Allergic Rhinitis Present *				0.33 ^c^
No	12 (35.3)	7 (43.8)	5 (27.8)	
Yes	22 (64.7)	9 (56.3)	13 (72.2)	
Diagnosed with AERD				0.10 ^d^
No	41 (93.2)	23 (100.0)	18 (85.7)	
Yes	3 (6.8)	0 (0.00)	3 (14.3)	
**Systemic Cytokines (pg/μL)**				
*Alarmins*				
IL-33 *	71.5 [12.0, 266.3]	17.0 [12.0, 243.2]	103.4 [36.4, 782.2]	0.54 ^b^
TSLP *	7.5 [1.8, 71.4]	2.3 [0.76, 24.5]	21.1 [4.5, 136.5]	0.33 ^b^
*Type 1 Disease Cytokines*				
IL-6 *	1.7 [0.60, 3.4]	2.3 [0.84, 3.7]	1.01 [0.49, 2.7]	0.32 ^b^
IL-8 *	2.4 [1.6, 3.5]	2.6 [1.5, 3.5]	2.4 [1.6, 3.7]	0.99 ^b^
IL-1 beta *	10.7 [4.0, 41.7]	16.3 [5.8, 46.0]	10.1 [3.8, 21.3]	0.45 ^b^
TNF-α *	29.8 [23.5, 47.6]	35.5 [26.3, 47.6]	26.4 [18.5, 40.1]	0.33 ^b^
*Type 2 Disease Cytokines*				
IL-5 *	9.7 [3.9, 18.2]	8.4 [3.8, 17.8]	11.9 [5.5, 25.0]	0.28 ^b^
IL-13 *	79.8 [63.0, 125.2]	95.4 [69.3, 143.1]	65.1 [27.8, 101.2]	0.19 ^b^
*Type 3 Disease Cytokines*				
IL-17A *	9.9 [2.2, 20.3]	12.2 [6.0, 22.5]	9.2 [0.96, 13.2]	0.35 ^b^
IL-17E/IL-25 *	1347.5 [515.9, 2849.2]	1225.9 [719.0, 2849.2]	1347.5 [475.0, 3210.1]	0.78 ^b^
**Nasal Cytokines (pg/μL)**				
*Alarmins*				
IL-33 *	450.8 [174.5, 833.1]	465.8 [148.2, 795.5]	428.0 [174.5, 1046.6]	0.76 ^b^
TSLP *	1.2 [0.56, 15.3]	6.0 [1.06, 19.9]	0.94 [0.38, 8.7]	0.32 ^b^
*Type 1 Disease Cytokines*				
TNF-α *	5.5 [3.7, 8.1]	5.1 [3.4, 8.5]	5.8 [4.3, 7.7]	0.79 ^b^
IL-6 *	12.4 [6.1, 54.2]	12.1 [5.5, 54.2]	14.5 [8.4, 61.4]	0.79 ^b^
IL-1 beta *	9.9 [3.4, 49.6]	16.7 [2.3, 49.6]	9.5 [4.5, 51.1]	0.96 ^b^
IL-8 *	867.3 [429.9, 1598.7]	912.0 [496.9, 1972.1]	785.4 [425.9, 1261.6]	0.55 ^b^
*Type 2 Disease Cytokines*				
IL-5 *	9.9 [3.4, 49.6]	16.7 [2.3, 49.6]	9.5 [4.5, 51.1]	0.96 ^b^
IL-13 *	30.0 [15.2, 57.9]	30.0 [17.4, 52.6]	31.1 [15.1, 85.0]	0.94 ^b^
*Type 3 Disease Cytokines*				
IL-17A *	1.8 [0.75, 6.3]	1.8 [0.75, 6.3]	1.2 [0.57, 11.0]	0.91 ^b^
IL-17E/IL-25 *	5.8 [2.7, 12.6]	7.7 [2.1, 12.9]	5.1 [3.1, 11.3]	0.93 ^b^

* Data not available for all subjects. Statistics presented as median [P25, P75], N (column %). *p*-values: ^b^ = Wilcoxon Rank Sum test, ^c^ = Pearson’s chi-square test, ^d^ = Fisher’s exact test.

**Table 2:** Differences in predictors between responder versus non-responder patients with chronic rhinosinusitis with nasal polyps.

Factor	Total (N = 26)	Non-Responder (N = 10)	Responder (N = 16)	*p*-Value
Age (years)	52.0 [37.0, 64.0]	62.5 [42.0, 67.0]	45.0 [33.5, 60.0]	0.17 ^b^
BMI (kg/m^2^)	28.3 [24.9, 39.2]	28.3 [27.8, 39.2]	28.1 [24.8, 36.2]	0.52 ^b^
Race *				0.99 ^d^
Non-White	2 (8.0)	1 (10.0)	1 (6.7)	
White	23 (92.0)	9 (90.0)	14 (93.3)	
Gender				0.99 ^d^
Male	18 (69.2)	7 (70.0)	11 (68.8)	
Female	8 (30.8)	3 (30.0)	5 (31.3)	
Type of Surgery				0.35 ^d^
Primary	20 (76.9)	9 (90.0)	11 (68.8)	
Revision	6 (23.1)	1 (10.0)	5 (31.3)	
Smoking Status				0.42 ^d^
Never	16 (61.5)	5 (50.0)	11 (68.8)	
Current or Former	10 (38.5)	5 (50.0)	5 (31.3)	
Asthma Present				0.11 ^d^
No	10 (38.5)	6 (60.0)	4 (25.0)	
Yes	16 (61.5)	4 (40.0)	12 (75.0)	
Allergic Rhinitis Present *				0.35 ^d^
No	8 (38.1)	4 (57.1)	4 (28.6)	
Yes	13 (61.9)	3 (42.9)	10 (71.4)	
Diagnosed with AERD				0.26 ^d^
No	23 (88.5)	10 (100.0)	13 (81.3)	
Yes	3 (11.5)	0 (0.00)	3 (18.8)	
**Systemic Cytokines (pg/μL)**				
*Alarmins*				
IL-33 *	96.3 [20.5, 1298.0]	20.5 [12.0, 1230.6]	697.2 [83.9, 2487.0]	0.26 ^b^
TSLP *	10.2 [6.4, 334.5]	7.5 [1.8, 334.5]	106.0 [8.3, 2983.5]	0.61 ^b^
*Type 1 Disease Cytokines*				
TNF-α *	6.2 [4.7, 8.5]	5.5 [3.1, 8.5]	6.7 [5.0, 8.1]	0.53 ^b^
IL-6 *	1.2 [0.60, 3.4]	2.6 [1.2, 3.7]	0.79 [0.59, 2.2]	0.32 ^b^
IL-8 *	2.9 [1.9, 4.3]	2.2 [1.5, 3.5]	3.3 [2.3, 4.9]	0.28 ^b^
IL-1 beta *	12.9 [5.7, 57.2]	46.0 [9.4, 68.3]	10.7 [1.04, 42.2]	0.35 ^b^
*Type 2 Disease Cytokines*				
IL-5 *	11.7 [4.6, 20.8]	9.4 [1.3, 10.0]	13.6 [7.1, 26.6]	0.17 ^b^
IL-13 *	64.4 [25.6, 145.4]	63.7 [51.6, 88.4]	65.1 [16.9, 213.8]	0.99 ^b^
*Type 3 Disease Cytokines*				
IL-17A *	3.3 [0.75, 11.0]	4.5 [0.75, 10.7]	3.0 [0.90, 14.0]	0.99 ^b^
IL-17E/IL-25 *	1159.9 [571.0, 5401.2]	932.6 [825.0, 2849.2]	1387.3 [475.0, 7953.3]	0.75 ^b^
**Nasal Cytokines (pg/μL)**				
*Alarmins*				
IL-33 *	318.9 [148.2, 975.7]	361.6 [148.2, 795.5]	318.9 [174.5, 1030.0]	0.86 ^b^
TSLP *	1.08 [0.38, 8.7]	1.3 [1.06, 10.8]	0.80 [0.35, 5.9]	0.49 ^b^
*Type 1 Disease Cytokines*				
IL-6 *	12.4 [7.5, 54.2]	12.3 [5.2, 31.9]	12.4 [9.1, 97.8]	0.46 ^b^
IL-8 *	703.5 [421.9, 1197.1]	713.7 [496.9, 1197.1]	703.5 [421.9, 873.6]	0.77 ^b^
IL-1 beta *	9.4 [5.7, 58.7]	15.6 [8.2, 43.8]	9.2 [5.7, 58.7]	0.95 ^b^
TNF-α *	6.2 [4.7, 8.5]	5.5 [3.1, 8.5]	6.7 [5.0, 8.1]	0.53 ^b^
*Type 2 Disease Cytokines*				
IL-5 *	28.1 [17.0, 116.9]	17.4 [16.6, 17.9]	62.6 [28.1, 117.9]	***0.037 ^b^***
IL-13 *	43.6 [17.4, 84.4]	26.9 [17.4, 46.4]	44.1 [18.3, 85.6]	0.33 ^b^
*Type 3 Disease Cytokines*				
IL-17E/IL-25 *	11.1 [2.3, 12.9]	12.9 [1.1, 12.9]	10.9 [4.8, 11.4]	0.99 ^b^
IL-17A *	3.3 [0.75, 11.0]	4.5 [0.75, 10.7]	3.0 [0.90, 14.0]	0.99 ^b^

* Data not available for all subjects. Statistics presented as median [P25, P75], N (column %). *p*-values: ^b^ = Wilcoxon Rank Sum test, ^d^ = Fisher’s exact test.

**Table 3:** Differences in Predictors Between Responder versus Non-responder Patients with Chronic Rhinosinusitis without Nasal Polyps.

Factor	Total (N = 18)	Non-Responder (N = 13)	Responder (N = 5)	*p*-Value
Age (years)	56.5 [38.0, 67.0]	48.0 [34.0, 67.0]	58.0 [55.0, 63.0]	0.53 ^b^
BMI (kg/m^2^)	31.4 [26.5, 34.5]	29.4 [26.5, 33.6]	34.5 [31.9, 35.1]	0.39 ^b^
Race *				0.99 ^d^
Non-White	3 (17.6)	2 (16.7)	1 (20.0)	
White	14 (82.4)	10 (83.3)	4 (80.0)	
Gender				0.60 ^d^
Male	7 (38.9)	6 (46.2)	1 (20.0)	
Female	11 (61.1)	7 (53.8)	4 (80.0)	
Type of Surgery				0.35 ^d^
Primary	20 (76.9)	9 (90.0)	11 (68.8)	
Revision	6 (23.1)	1 (10.0)	5 (31.3)	
Smoking Status				0.25 ^d^
Never	13 (72.2)	8 (61.5)	5 (100.0)	
Current or Former	5 (27.8)	5 (38.5)	0 (0.00)	
Asthma Present				0.61 ^d^
No	6 (33.3)	5 (38.5)	1 (20.0)	
Yes	12 (66.7)	8 (61.5)	4 (80.0)	
Allergic Rhinitis Present *				0.99 ^d^
No	4 (30.8)	3 (33.3)	1 (25.0)	
Yes	9 (69.2)	6 (66.7)	3 (75.0)	
Diagnosed with AERD				
No	18 (100.0)	13 (100.0)	5 (100.0)	
**Systemic Cytokines (pg/μL)**				
*Alarmins*				
IL-33 *	14.5 [7.7, 243.2]	14.5 [11.5, 243.2]	55.9 [0.69, 188.4]	0.61 ^b^
TSLP *	2.5 [0.71, 32.0]	1.5 [0.71, 24.5]	17.4 [1.4, 51.7]	0.76 ^b^
*Type 1 Disease Cytokines*				
IL-6 *	2.3 [0.41, 3.4]	2.2 [0.72, 3.8]	2.3 [0.39, 3.1]	0.72 ^b^
IL-8 *	2.1 [0.91, 3.3]	3.1 [1.6, 3.3]	1.6 [0.91, 2.4]	0.25 ^b^
IL-1 beta *	9.4 [3.5, 27.0]	11.0 [2.2, 34.6]	9.4 [4.6, 15.7]	0.94 ^b^
TNF-α *	29.8 [23.6, 47.6]	38.3 [29.0, 47.6]	23.6 [18.5, 30.0]	0.31 ^b^
*Type 2 Disease Cytokines*				
IL-5 *	8.4 [3.9, 17.8]	7.4 [4.8, 17.8]	10.2 [3.9, 10.2]	0.99 ^b^
IL-13 *	101.2 [70.6, 125.2]	110.1 [82.3, 143.1]	67.5 [53.9, 86.6]	0.078 ^b^
*Type 3 Disease Cytokines*				
IL-17A *	9.6 [6.0, 13.7]	8.8 [3.7, 21.4]	10.2 [7.2, 12.6]	0.94 ^b^
IL-17E/IL-25 *	1413.5 [515.9, 1884.6]	1519.2 [719.0, 1884.6]	1307.7 [515.9, 1571.8]	0.79 ^b^
**Nasal Cytokines (pg/μL)**				
*Alarmins*				
IL-33 *	698.8 [196.6, 833.1]	642.2 [196.6, 707.4]	873.2 [699.9, 1046.6]	0.31 ^b^
TSLP *	19.9 [0.80, 24.0]	19.9 [0.60, 34.4]	12.4 [0.80, 24.0]	0.99 ^b^
*Type 1 Disease Cytokines*				
IL-6 *	14.4 [5.8, 53.6]	12.1 [6.1, 87.0]	16.6 [0.77, 20.2]	0.72 ^b^
IL-8 *	1040.8 [651.6, 2096.4]	1157.1 [694.1, 2220.8]	924.5 [609.0, 1947.1]	0.72 ^b^
IL-1 beta *	9.4 [3.5, 27.0]	11.0 [2.2, 34.6]	9.4 [4.6, 15.7]	0.94 ^b^
TNF-α *	4.9 [3.4, 6.1]	5.1 [3.7, 6.5]	3.3 [1.8, 5.5]	0.22 ^b^
*Type 2 Disease Cytokines*				
IL-5 *	9.2 [4.5, 31.6]	10.2 [7.5, 49.5]	7.7 [0.75, 13.7]	0.48 ^b^
IL-13 *	21.9 [14.7, 43.8]	30.0 [21.0, 52.6]	15.0 [12.1, 18.6]	0.12 ^b^
*Type 3 Disease Cytokines*				
IL-17A *	1.8 [0.57, 3.6]	1.8 [1.2, 4.2]	1.00 [0.16, 3.4]	0.49 ^b^
IL-17E/IL-25 *	4.3 [3.1, 8.9]	6.4 [3.1, 12.4]	3.9 [2.2, 4.7]	0.51 ^b^

* Data not available for all subjects. Statistics presented as median [P25, P75], N (column %). *p*-values: ^b^ = Wilcoxon Rank Sum test, ^d^ = Fisher’s exact test.

## Data Availability

The data presented in this study is available in the article.

## Supplementary Materials

The following supporting information can be downloaded at: https://www.mdpi.com/article/10.3390/jcm14134446/s1, Table 1. Missing in Predictors Between Responder Groups—All Patients; Table S2. Missing in Predictors Between Responder Groups—CRSwNP; Table S3. Missing in Predictors Between Responder Groups—CRSsNP.
