# Supplementary material for: Investigating the Role of the Sinus Microbiome and Cytokine Profile in the SNOT-22 Response After Functional Endoscopic Sinus Surgery in Chronic Rhinosinusitis Patients
Source: J Clin Med. 2025 Jun 23;14(13):4446. doi: 10.3390/jcm14134446 (PMC12250471; doi:10.3390/jcm14134446)
Supplement: Supplementary file 1 [file jcm-14-04446-s001.zip › jcm-3670369-supplementary.pdf]

Supplementary Table S1. Missing in Predictors Between Responder Groups—All Patients

Factor	Overall (N=44)	Non-Responder (N=23)	Responder (N=21)
Age	0(0.0)	0(0.0)	0(0.0)
BMI (kg/m)	0(0.0)	0(0.0)	0(0.0)
Race	2(4.5)	1(4.3)	1(4.8)
Gender	0(0.0)	0(0.0)	0(0.0)
Study Group	0(0.0)	0(0.0)	0(0.0)
Type of Surgery	0(0.0)	0(0.0)	0(0.0)
Smoking Status	0(0.0)	0(0.0)	0(0.0)
Asthma Present	0(0.0)	0(0.0)	0(0.0)
Allergic Rhinitis Present	10(22.7)	7(30.4)	3(14.3)
Diagnosed with AERD	0(0.0)	0(0.0)	0(0.0)
**Systemic Cytokines (pg/μL)**			
Alarmins			
IL-33	27(61.4)	14(60.9)	13(61.9)
TSLP	27(61.4)	14(60.9)	13(61.9)
Type 1 Disease Cytokines			
IL-6	20(45.5)	11(47.8)	9(42.9)
IL-8	18(40.9)	9(39.1)	9(42.9)
IL-1 beta	19(43.2)	10(43.5)	9(42.9)
TNF- α	18(40.9)	9(39.1)	9(42.9)
Type 2 Disease Cytokines			
IL-5	18(40.9)	9(39.1)	9(42.9)
IL-13	19(43.2)	9(39.1)	10(47.6)
Type 3 Disease Cytokines			
IL-17A	19(43.2)	10(43.5)	9(42.9)
IL-17E/IL-25	18(40.9)	9(39.1)	9(42.9)
**Nasal Cytokines (pg/μL)**			
Alarmins			
IL-33	18(40.9)	8(34.8)	10(47.6)
TSLP	28(63.6)	17(73.9)	11(52.4)
Type 1 Disease Cytokines			
IL-6	17(38.6)	8(34.8)	9(42.9)
IL-8	17(38.6)	8(34.8)	9(42.9)
IL-1 beta	17(38.6)	8(34.8)	9(42.9)
TNF- α	17(38.6)	8(34.8)	9(42.9)
Type 2 Disease Cytokines			
IL-5	17(38.6)	8(34.8)	9(42.9)
IL-13	17(38.6)	8(34.8)	9(42.9)
Type 3 Disease Cytokines			
IL-17A	19(43.2)	9(39.1)	10(47.6)
IL-17E/IL-25	20(45.5)	11(47.8)	9(42.9)

Missing values presented as frequency (percent)

Supplementary Table S2. Missing in Predictors Between Responder Groups—CRSwNP

Factor	Overall (N=26)	Non-Responder (N=10)	Responder (N=16)
Age	0(0.0)	0(0.0)	0(0.0)
BMI (kg/m)	0(0.0)	0(0.0)	0(0.0)
Race	1(3.8)	0(0.0)	1(6.3)
Gender	0(0.0)	0(0.0)	0(0.0)
Type of Surgery	0(0.0)	0(0.0)	0(0.0)
Smoking Status	0(0.0)	0(0.0)	0(0.0)
Asthma Present	0(0.0)	0(0.0)	0(0.0)
Allergic Rhinitis Present	5(19.2)	3(30.0)	2(12.5)
Diagnosed with AERD	0(0.0)	0(0.0)	0(0.0)
**Systemic Cytokines (pg/µL)**			
Alarmins			
IL-33	19(73.1)	7(70.0)	12(75.0)
TSLP	19(73.1)	7(70.0)	12(75.0)
Type 1 Disease Cytokines			
IL-6	15(57.7)	6(60.0)	9(56.3)
IL-8	14(53.8)	5(50.0)	9(56.3)
IL-1 beta	14(53.8)	5(50.0)	9(56.3)
TNF-α	14(53.8)	5(50.0)	9(56.3)
Type 2 Disease Cytokines			
IL-5	14(53.8)	5(50.0)	9(56.3)
IL-13	14(53.8)	5(50.0)	9(56.3)
Type 3 Disease Cytokines			
IL-17A	14(53.8)	5(50.0)	9(56.3)
IL-17E/IL-25	14(53.8)	5(50.0)	9(56.3)
**Nasal Cytokines (pg/µL)**			
Alarmins			
IL-33	11(42.3)	4(40.0)	7(43.8)
TSLP	15(57.7)	7(70.0)	8(50.0)
Type 1 Disease Cytokines			
IL-6	11(42.3)	4(40.0)	7(43.8)
IL-8	11(42.3)	4(40.0)	7(43.8)
IL-1 beta	11(42.3)	4(40.0)	7(43.8)
TNF-α	11(42.3)	4(40.0)	7(43.8)
Type 2 Disease Cytokines			
IL-5	11(42.3)	4(40.0)	7(43.8)
IL-13	11(42.3)	4(40.0)	7(43.8)
Type 3 Disease Cytokines			
IL-17A	12(46.2)	4(40.0)	8(50.0)
IL-17E/IL-25	12(46.2)	5(50.0)	7(43.8)

Missing values presented as frequency (percent)

Supplementary Table S3. Missing in Predictors Between Responder Groups—CRSsNP

Factor	Overall (N=18)	Non-Responder (N=13)	Responder (N=5)
Age	0(0.0)	0(0.0)	0(0.0)
BMI (kg/m)	0(0.0)	0(0.0)	0(0.0)
Race	1(5.6)	1(7.7)	0(0.0)
Gender	0(0.0)	0(0.0)	0(0.0)
Type of Surgery	0(0.0)	0(0.0)	0(0.0)
Smoking Status	0(0.0)	0(0.0)	0(0.0)
Asthma Present	0(0.0)	0(0.0)	0(0.0)
Allergic Rhinitis Present	5(27.8)	4(30.8)	1(20.0)
Diagnosed with AERD	0(0.0)	0(0.0)	0(0.0)
**Systemic Cytokines (pg/µL)**			
Alarmins			
IL-33	8(44.4)	7(53.8)	1(20.0)
TSLP	8(44.4)	7(53.8)	1(20.0)
Type 1 Disease Cytokines			
IL-6	5(27.8)	5(38.5)	0(0.0)
IL-8	4(22.2)	4(30.8)	0(0.0)
IL-1 beta	5(27.8)	5(38.5)	0(0.0)
TNF alpha	4(22.2)	4(30.8)	0(0.0)
Type 2 Disease Cytokines			
IL-5	4(22.2)	4(30.8)	0(0.0)
IL-13	5(27.8)	4(30.8)	1(20.0)
Type 3 Disease Cytokines			
IL-17A	5(27.8)	5(38.5)	0(0.0)
IL-17E/IL-25	4(22.2)	4(30.8)	0(0.0)
**Nasal Cytokines (pg/µL)**			
Alarmins			
IL-33	7(38.9)	4(30.8)	3(60.0)
TSLP	13(72.2)	10(76.9)	3(60.0)
Type 1 Disease Cytokines			
IL-6	6(33.3)	4(30.8)	2(40.0)
IL-8	6(33.3)	4(30.8)	2(40.0)
IL-1 beta	6(33.3)	4(30.8)	2(40.0)
TNF alpha	6(33.3)	4(30.8)	2(40.0)
Type 2 Disease Cytokines			
IL-5	6(33.3)	4(30.8)	2(40.0)
IL-13	6(33.3)	4(30.8)	2(40.0)
Type 3 Disease Cytokines			
IL-17A	7(38.9)	5(38.5)	2(40.0)
IL-17E/IL-25	8(44.4)	6(46.2)	2(40.0)

Missing values presented as frequency (percent)
